# Virtuous personality and bystander defending behavior among college students: roles of moral identity and friendship quality

**DOI:** 10.1186/s40359-025-03058-4

**Published:** 2025-07-04

**Authors:** Shen Liu, Ping Wang, Wenxiao Gao, Minghua Song, Na Zhang

**Affiliations:** 1 Department of Psychology, School of Humanities and Social Sciences, Anhui Agricultural University, Hefei, 230036 China; 2 Mental Health Education Guidance Center, Huzhou University, Huzhou, China; 3 Department of Information Management, Anhui Vocational College of Police Officers, Hefei, 230036 China

**Keywords:** Virtuous personality, Bystander defending behavior, Moral identity, Friendship quality, College students

## Abstract

**Background:**

To explore the relationship between virtuous personality and bystander defending behavior among college students, and to examine the roles of moral identity and friendship quality, a survey was conducted with 643 college students.

**Methods:**

The survey used the Chinese Virtuous Personality Scale, the Bystander Defending Behavior Scale, the Moral Identity Scale, and the Friendship Quality Inventory. To test the hypotheses, descriptive statistics, *t*-tests, and correlation analysis were conducted using SPSS 22.0. Additionally, mediation and moderation effects were tested using the SPSS macro program PROCESS, specifically Models 4, 7, and 59.

**Results:**

The results revealed the following: (1) a significant positive correlation was found between virtuous personality, bystander defending behavior, moral identity, and friendship quality; (2) virtuous personality not only directly and positively predicted bystander defending behavior, but also influenced this behavior through the mediating role of moral identity; (3) the first part of the mediating effect— “virtuous personality → moral identity → bystander defending behavior”—was moderated by friendship quality. Specifically, the impact of virtuous personality on moral identity was stronger in individuals with higher friendship quality compared to those with lower friendship quality.

**Conclusions:**

These findings suggest that virtuous personality can enhance bystander defending behavior among college students by fostering moral identity. However, lower levels of friendship quality can weaken the positive influence of virtuous personality on moral identity, thereby affecting bystander defending behavior.

## Introduction

Bullying is a common phenomenon in everyday life. Whether it occurs in schools, online, or in the workplace, bystanders are nearly always present, and their numbers far exceed those of both the bullies and the victims [1]. As witnesses, informants, and key third parties in bullying incidents, bystanders’ psychological tendencies and behaviors, as well as their attitudes toward both the bullies and the victims, play a significant role in facilitating the occurrence and escalation of bullying [2]. In bullying incidents, based on the different behaviors and reactions of bystanders, Salmivalli et al. [3] classified bystanders into reinforcers, assistants, protectors, and outsiders. Protectors are often characterized by their proactive and selfless efforts to help the victim, which is the focus of this study. Bystander defending behavior refers to individuals actively supporting or assisting the victim, or directly opposing the bully, in an effort to stop the bullying [4]. This behavior plays a crucial role in curbing the occurrence of bullying. For example, when bystanders engage in protective behavior, about 57% of bullies will stop their bullying within ten seconds [5]. When victims receive intervention, it helps reduce their pain and harm [6]. Youth are the pillars of a nation—“As the youth grow strong, the nation grows strong.” As a key part of the new generation, college students play an important role. Therefore, exploring the factors that influence bystander defending behavior among college students and revealing its underlying mechanisms is crucial for reducing and preventing bullying incidents.

### Virtuous personality and bystander defending behavior

Throughout history, diligence and virtuous personality have consistently been regarded as core virtues. In the field of personality psychology, the concept of a " virtuous personality " refers to the positive traits that individuals gradually develop and outwardly exhibit in their interactions with others, such as responsibility, honesty, friendliness, and altruism [7]– [8]. According to social cognitive theory of personality, an individual’s behavioral responses are also linked to their cognitive processes in specific situations [9]. Specifically, moral cognition, self-efficacy, and outcome expectancies interact to influence an individual’s response to bullying. An individual’s cognitive processing of different levels of bullying situations, particularly their moral evaluation of the situation, their assessment of their ability to intervene effectively (self-efficacy), and their belief in the positive consequences of intervention (outcome expectancies), is an important factor in triggering bystander defending behavior [10]– [11]. For example, even if an individual recognizes the moral wrongness of bullying, they may not intervene if they lack self-efficacy or believe that their intervention will be ineffective [12]. Research has found that individuals with higher levels of virtuous personality traits not only focus positively on the positive emotional information in interpersonal relationships, but also effectively manage and mitigate negative emotions, taking proactive actions to maintain good relationships [13]. Other studies have shown that virtuous personality traits influence an individual’s subjective well-being [14], significantly positively impact altruistic behaviors [15], including altruistic behavior in online environments [8], and have a bidirectional predictive relationship with the satisfaction of moral needs [14]. Bystander defending behavior, on the other hand, refers to the proactive behavioral response of a protector when facing a bullying situation, and it is considered a form of prosocial behavior [16]. Based on these findings, it can be inferred that individuals with a virtuous personality are able to manage and alleviate negative emotions in bullying situations and take positive actions to maintain interpersonal relationships. Therefore, this study proposes Hypothesis *H*_1_: Virtuous personality positively predicts bystander defending behavior.

### The mediating role of moral identity

Moral identity was first defined as an individual difference that reflects the extent to which morality is a central or defining feature of one’s self-awareness [17]. Moral identity refers to the self-conceptual framework built around a set of morally related traits, such as honesty, and care, and includes two dimensions: internalization and representation [17]. Research has shown that moral identity can significantly predict prosocial behaviors among college students [18] and online altruistic behaviors [17]. When an individual’s self-concept is centered around a particular moral identity, this tendency becomes a key internal drive for engaging in moral behavior [19]. Individuals with high moral identity tend to feel discomfort when others are harmed, which increases their willingness to help and fosters bystander defending behaviors such as stopping the bully or notifying authorities in online environments [20]– [21]. In contrast, individuals with low moral identity exhibit fewer prosocial behaviors [22]. Existing studies have also shown that moral identity can significantly and positively predict bystander defending behavior [16]. Moral identity, as a self-conceptual framework, develops through an individual’s interactions and connections with others over time, with different social environments influencing the formation of this framework [17]. From a developmental perspective, sound personality traits play an important role in the formation of students’ moral identity, and personality variables cannot be ignored in predicting moral behavior [23]. Concepts of benevolence and moral teachings have long played a crucial role in the formation and development of moral identity across various cultural contexts. Research has shown that individuals with higher levels of virtuous personality tend to have a stronger moral need for fulfillment [14]. Personality traits differ across individuals, and people adopt various strategies to fulfill their needs. Research has shown that individuals with a higher level of virtuous personality tend to have greater moral needs, which, in turn, enables them to translate their moral understanding into practical actions and behave morally [14]. Individuals with a strong moral identity are more likely to volunteer proactively and donate more than others [23]– [24]. Within this framework, it can be inferred that moral identity serves as a critical mediator through which virtuous personality traits affect bystander defending behavior. In other words, individuals with higher levels of virtuous personality are more likely to internalize a strong moral identity, which motivates them to engage in prosocial actions, such as intervening in situations where assistance is needed. Therefore, this study proposes Hypothesis *H*_2_: Moral identity mediates the relationship between virtuous personality and bystander defending behavior.

### The moderating effect of friendship quality

While virtuous personality traits influence bystander defending behavior through moral identity, they may also be moderated by other variables. As college students age and gain more life experience, their surrounding environment and interpersonal relationships undergo changes, with friendship beginning to play an increasingly important role in their social lives during early adulthood. For example, friendship can help individuals gain more social support, improve self-esteem and self-identity, reduce negative emotions such as anxiety and depression, and promote both psychological well-being and social development [25]– [26]. Friendship is a key dimension of peer relationships, and friendship quality refers to the level of mutual companionship, support, and conflict between individuals and their friends. It can reflect an individual’s social skills to some extent [27] and is also an important indicator of social adaptation in the process of social development [16]. For college students, the transition from adolescence to early adulthood, coupled with the sudden increase in academic pressure, can lead to feelings of confusion and anxiety. In this context, the quality of friendship plays a crucial role in alleviating these feelings and has a significant impact on the individual’s psychological and behavioral development. Alsarrani et al. [28] found that individuals with higher friendship quality are more likely to exhibit prosocial behaviors. While both virtuous personality and moral identity influence college students’ bystander defending behavior, these effects may vary depending on individual differences. According to the theory of individual-environment interaction, the development of an individual’s psychological tendencies and behavioral attitudes is also shaped by the interaction between the individual and their environment [25]. Peer relationships, as part of the external environment, certainly influence an individual’s protective behavior as a bystander, but the degree of influence is closely related to the individual’s own qualities. Friendship quality, as a key indicator of peer relationships, plays an important role in this process. Based on this, the present study proposes Hypothesis *H*_3a_: Friendship quality moderates the relationship between virtuous personality and bystander defending behavior. Good friendship quality plays a crucial protective role in the psychological and behavioral development of individuals, positively promoting their healthy growth. Previous research has shown that friendship quality indirectly influences individual behavior by affecting cognitive, emotional, and other aspects, thus moderating the development of personality and behavior, and enhancing psychological well-being [29]– [30]. Moral identity is an important aspect of college students’ intrinsic self-concept, and it can be inferred that friendship quality may also influence this factor, subsequently affecting bystander defending behavior. Based on this, the present study proposes Hypothesis *H*_3b_: Friendship quality moderates the relationship between virtuous personality and moral identity. Furthermore, positive peer relationships help enhance an individual’s perception of social support, thereby strengthening their moral identity [31], which in turn promotes bystander defending behavior. It is therefore hypothesized that for college students with high friendship quality, a higher level of moral identity will lead to a greater willingness to help, increasing bystander defending behaviors such as stopping the bully and notifying authorities in online environments. Based on this, the study proposes Hypothesis *H*_3c_: Friendship quality moderates the relationship between moral identity and bystander defending behavior.

**Fig. 1:**
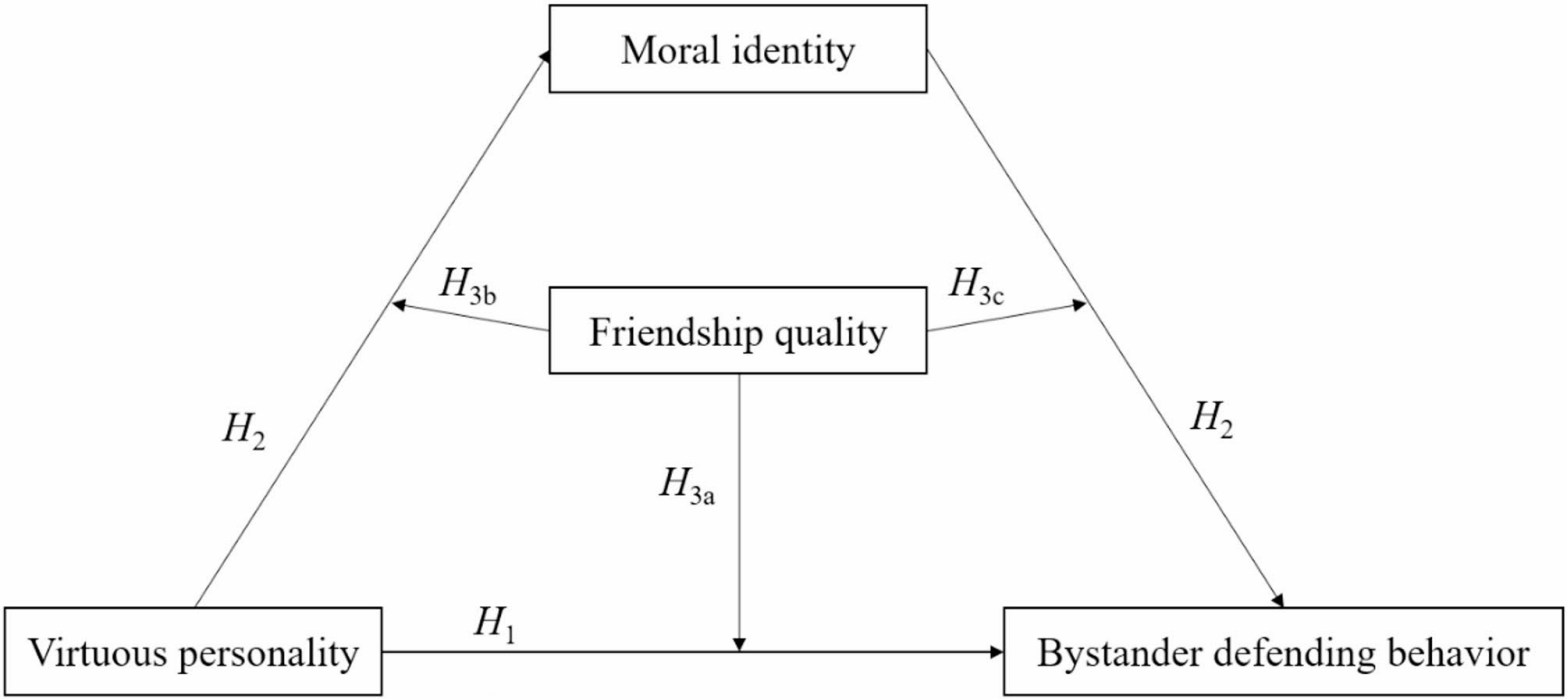
Theoretical model

In summary, this study, based on social-cognitive theory of personality, integrates moral identity theory and the theory of individual-environment interaction. It focuses on college students to examine the influence of virtuous personality on bystander defending behavior, as well as the roles played by moral identity and friendship quality. The goal is to provide empirical evidence and intervention insights for addressing bullying incidents among college students. Accordingly, this study constructs a moderated mediation model, as shown in Fig. 1.

## Methods

### Participants

Using G*Power version 3.1.9.7, a priori power analysis was conducted [32]. The minimum sample size was calculated for the proposed mediation model of the present study. The parameters indicated were a medium effect size of 0.15 (Effect size *f*^2^ = 0.15) with α = 0.05, minimum Power (1 − *β*) = 0.95, and number of predictors = 3. In this case, a minimum sample of 120 participants was necessary. Thus, we used convenience sampling and distributed a total of 700 online and offline questionnaires across five universities in China. After screening and removing invalid responses, 643 valid entries remained, resulting in a response rate of 91.86%. Extreme values were identified through outlier analysis, with any responses exceeding 3 standard deviations from the mean excluded. Among the participants, 278 were male and 365 were female; 184 were only children, while 459 had siblings; 396 were from urban areas, and 247 were from rural areas. The average age of the participants was 19.65 years, with a standard deviation of 1.23. The study was approved by the Ethics Committee of the author’s institution, and all participants provided informed consent before completing the questionnaires.

### Measures

#### Chinese virtuous personality scale

The Chinese Virtuous Personality Scale, developed by Jiao et al. [33], was used in this study. The scale consists of four dimensions: altruistic dedication, tolerance, responsibility and integrity, and benevolence and friendliness, with a total of 15 items. Example items include: “When helping others, I rarely consider my own interests.” A 5-point Likert scale was employed, with 1 indicating “strongly disagree” and 5 indicating “strongly agree.” Higher scores reflect a higher level of virtuous personality. In this study, the internal consistency coefficient of the scale was 0.82. The results of the confirmatory factor analysis indicate good structural validity of the questionnaire: *χ*^²^/*df* = 3.86, CFI = 0.92, NFI = 0.94, GFI = 0.92, RMSEA = 0.003.

#### Moral identity scale

The Moral Identity Scale, developed by Aquino and Reed [34], was used in this study. The scale consists of two dimensions: externalization and internalization, with a total of 10 items. An example item is: " I rarely consider my own interests when helping others.” A 5-point Likert scale was used, with 1 indicating “strongly disagree” and 5 indicating “strongly agree.” Higher scores reflect a higher level of moral identity. In this study, the internal consistency coefficient of the scale was 0.80. The results of the confirmatory factor analysis indicate good structural validity of the questionnaire: *χ*^²^/*df* = 4.33, CFI = 0.93, NFI = 0.93, GFI = 0.92, RMSEA = 0.002.

#### Friendship quality inventory

The Friendship Quality Inventory (FQI), revised by Fan and Fang [35], was used in this study. The scale includes five dimensions: help and support, companionship conflict, partnership, intimacy, and conflict intensity, with a total of 15 items. An example item is: “When you need to accomplish something, your friends will come to help you.” A 5-point Likert scale was employed, with 1 indicating “never” and 5 indicating “always.” Higher scores reflect higher friendship quality. In this study, the internal consistency coefficient of the scale was 0.83. The results of the confirmatory factor analysis indicate good structural validity of the questionnaire: *χ*^²^/*df* = 3.88, CFI = 0.91, NFI = 0.90, GFI = 0.92, RMSEA = 0.003.

#### Bystander defending behavior scale

The Bystander Defending Behavior Scale, developed by Teng [36], includes three dimensions: protective behavior, pro-bullying behavior, and outsider behavior. This scale is designed to measure bystander defending behavior. For this study, only the defending behavior dimension was utilized, with scenario settings like, “If you were witnessing cyberbullying, as a bystander, how would you usually respond?” The scale includes 9 items. An example item is: “I would persuade the perpetrator or other potential participants to stop their behavior.” A 7-point Likert scale was used, with 1 indicating “strongly disagree” and 7 indicating “strongly agree.” Higher scores indicate a stronger tendency to engage in protective behaviors. In this study, the internal consistency coefficient of the scale was 0.88. The results of the confirmatory factor analysis indicate good structural validity of the questionnaire: *χ*^²^/*df* = 3.22, CFI = 0.93, NFI = 0.93, GFI = 0.92, RMSEA = 0.002.

### Data processing

Descriptive statistics, t-tests, and correlation analysis were conducted using SPSS 22.0. Additionally, mediation and moderation effects were tested using the SPSS macro program PROCESS [37], specifically Models 4, 7, and 59 (downloaded from http://www.afhayes.com).

## Results

### Common methods bias testing

To minimize potential common method bias, this study implemented procedural controls to reduce its impact, such as having participants complete the questionnaires anonymously and using reverse-scored items in the questionnaire design. To further enhance scientific rigor, Harman’s single-factor test was conducted to examine common method bias across all items of the scales used. The results showed that there were 11 factors with eigenvalues greater than 1, with the first extracted factor explaining 22.61% of the variance, which is below the 40% critical threshold, indicating that there is no significant common method bias in this study [38].

### Descriptive statistics and correlation analysis

The descriptive statistics and correlation analysis results for each variable are presented in Table 1. Among them, virtuous personality is significantly positively correlated with moral identity, friendship quality, and bystander defending behavior; moral identity is significantly positively correlated with both friendship quality and bystander defending behavior; and friendship quality is significantly positively correlated with bystander defending behavior. These results suggest that the data in this study are suitable for subsequent analyses.

**Table 1:** Correlation matrices of variables (*n* = 643)

Variables	M	SD	1	2	3	4	5	6	7
1. Gender	–	–							
2. Only children or not	–	–	0.14^**^						
3. Permanent residence	–	–	0.06	0.21^**^					
4. Virtuous personality	3.40	0.55	0.09^*^	-0.05	-0.01	–			
5. Moral identity	3.76	0.53	0.08^*^	-0.04	-0.07	0.52^**^	–		
6. Friendship quality	3.62	0.52	0.18^*^	-0.09^*^	-0.07	0.37^**^	0.46^*^	–	
7. bystander defending behavior	5.28	0.98	0.08^*^	-0.04	-0.02	0.52^**^	0.51^**^	0.44^**^	–

Note: ^*^p-value < 0.05, ^**^p-value < 0.01, and ^***^p-value < 0.001, the same as below

### Moderated mediation effect analysis

The independent samples t-test results indicate significant gender differences in virtuous personality (*t* = 2.21, *p* < 0.05), moral identity (*t* = 2.11, *p* < 0.05), and friendship quality (*t* = 4.74, *p* < 0.05), with females showing significantly higher levels of virtuous personality, moral identity, and friendship quality than males. There is also a difference in friendship quality based on whether the individual is an only child, with only children having significantly higher friendship quality than non-only children (*t* = 2.16, *p* < 0.05). Furthermore, being an only child is significantly correlated with whether the individual resides in an urban or rural area. Therefore, gender, whether the individual is an only child, and place of residence will be treated as control variables in subsequent analyses. Based on the perspectives of Hayes [37] and Wen and Ye [39], the SPSS macro program PROCESS Model 4 was first used to test the mediating role of moral identity in the relationship between virtuous personality and bystander defending behavior. The results showed that, after controlling for gender, place of residence, and whether the individual is an only child, virtuous personality significantly and positively predicted moral identity (*β* = 0.51, 95%CI=[LLCI = 0.45, ULCI = 0.58], *p* < 0.001). When both virtuous personality and moral identity were included in the regression equation, virtuous personality (*β* = 0.35, 95%CI=[LLCI = 0.28, ULCI = 0.42], *p* < 0.001) and moral identity (*β* = 0.33, 95%CI=[LLCI = 0.26, ULCI = 0.40], *p* < 0.001) both significantly and positively predicted bystander defending behavior. The bias-corrected percentile Bootstrap test indicated that moral identity significantly mediated the relationship between virtuous personality and bystander defending behavior (*β* = 0.17, 95%CI=[LLCI = 0.12, ULCI = 0.22], *p* < 0.001), with the mediating effect accounting for 32.69% of the total effect (*β* = 0.52, 95%CI=[LLCI = 0.45, ULCI = 0.59], *p* < 0.001).

Next, using Model 59 from PROCESS (assuming that the first half, second half, and direct path of the mediation model are moderated, consistent with the theoretical model of this study), analyze whether friendship quality moderates the relationship between virtuous personality, moral identity, and bystander defending behavior. For bystander defending behavior, as shown in Table 2, the predictive effect of virtuous personality on moral identity is significant (*β* = 0.41, 95%CI=[LLCI = 0.34, ULCI = 0.48], *p* < 0.001), and the interaction between virtuous personality and friendship quality has a significant predictive effect on moral identity (*β* = 0.05, 95%CI=[LLCI = 0.001, ULCI = 0.09], *p* = 0.047). Moreover, moral identity has a significant predictive effect on bystander defending behavior (*β* = 0.26, 95%CI=[LLCI = 0.19, ULCI = 0.34], *p* < 0.001). Therefore, the first half of the mediating effect of moral identity on virtuous personality is moderated by the quality of friendship. The interaction between virtuous personality and friendship quality had no significant predictive effect on bystander defending behavior (*β*=–0.04, 95%CI=[LLCI=–0.10, ULCI = 0.01], *p* = 0.117), and the interaction between moral identity and friendship quality had no significant predictive effect on bystander defending behavior (*β*=–0.02, 95%CI=[LLCI=–0.09, ULCI = 0.05], *p* = 0.565). Therefore, both the direct and secondary pathways of the mediating effect of virtuous personality on bystander defending behavior through moral identity are not moderated by the quality of friendship.

**Table 2:** Moderated mediation modeling testing

Regression equations		Overall fit index			Significance of regression coefficient		
Outcome variables	Predictive variables	*R*	*R* ^2^	*F*	*β*	95%CI	*t*
bystander defending behavior	Gender	0.59	0.35	56.18^***^	–0.01	[–0.08, 0.05]	–0.45
	Only children or not				0.004	[–0.06, 0.07]	0.14
	Permanent residence				0.01	[–0.06, 0.07]	0.23
	Virtuous personality				0.41	[0.34, 0.48]	11.79^***^
	Friendship quality				0.28	[0.21, 0.35]	7.99^***^
	Virtuous personality ×Friendship quality				–0.04	[–0.08, 0.01]	–1.66
Moral identity	Gender	0.60	0.36	58.88^***^	–0.01	[–0.07, 0.06]	–0.19
	Only children or not				0.03	[–0.04, 0.09]	0.76
	Permanent residence				–0.01	[–0.12, 0.01]	–1.72
	Virtuous personality				0.41	[0.34, 0.48]	11.92^***^
	Friendship quality				0.31	[0.25, 0.38]	8.95^***^
	Virtuous personality ×Friendship quality				0.05	[0.001, 0.09]	1.99^*^
Bystander defending behavior	Gender	0.63	0.39	50.96^***^	–0.01	[–0.07, 0.05]	–0.37
	Only children or not				–0.001	[–0.06, 0.06]	–0.04
	Permanent residence				0.02	[–0.04, 0.09]	0.71
	Virtuous personality				0.30	[0.23, 0.38]	8.12^***^
	Moral identity				0.26	[0.19, 0.34]	6.80^***^
	Friendship quality				0.20	[0.13, 0.27]	5.53^***^
	Virtuous personality ×Friendship quality				–0.04	[–0.10, 0.01]	–1.57
	Friendship quality ×Moral identity				–0.02	[–0.09, 0.05]	–0.58

Note: *The VIF values of virtuous personality*,* moral identity and friendship quality were 1.41*,* 1.54 and 1.31*,* respectively*

Using PROCESS Model 7 in SPSS (which assumes that the first half of the mediation path is moderated), we tested whether the mediation effect changes with variations in the moderator variable. The results indicated that at both levels of friendship quality—above (value = 0.27, *SE* = 0.04, 95%CI=[LLCI = 0.20, ULCI = 0.34]) and below (value = 0.22, *SE* = 0.05, 95%CI=[LLCI = 0.14, ULCI = 0.33]) one standard deviation—virtuous personality significantly mediated the effect on bystander defending behavior (with confidence intervals not including zero). However, the effect size varied between the two levels.

**Fig. 2:**
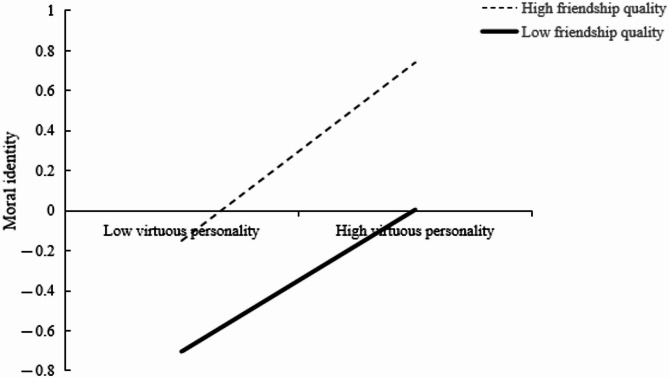
The moderating effect of friendship quality on the influence of virtuous personality on moral identity

To further explore how friendship quality moderates the effect of virtuous personality on moral identity, we divided friendship quality into high and low groups (± one standard deviation) and conducted simple slope analysis. The results showed that for low friendship quality (*M*–1*SD*), virtuous personality significantly and positively predicted moral identity (*B*_simple_=0.22, *p* < 0.001, 95%CI=[LLCI = 0.14, ULCI = 0.33]). For high friendship quality (*M* + 1*SD*), the positive prediction of virtuous personality on moral identity remained significant and was strengthened (*B*_simple_=0.27, *p* < 0.001, 95%CI=[LLCI = 0.20, ULCI = 0.34]) (see Fig. 2). This suggests that friendship quality enhances the effect of virtuous personality on moral identity.

## Discussion

This study explores the relationship between virtuous personality and bystander defending behavior among college students, as well as the underlying mechanisms, aiming to provide theoretical guidance and empirical evidence for preventing and reducing bullying incidents. The findings reveal that virtuous personality significantly and positively predicts bystander defending behavior in college students, with moral identity serving as a mediator in the relationship between virtuous personality and bystander defending behavior. Furthermore, the first half of this indirect effect is moderated by friendship quality.

This study found that virtuous personality significantly and positively predicts bystander defending behavior among college students, which is consistent with existing research [40]. This suggests that the higher the level of virtuous personality in college students, the more likely they are to engage in bystander defending behavior. Virtuous personality is typically characterized by traits such as empathy, generosity, and concern for others, making virtuous individuals more attuned to the emotions of others. They are able to empathize with the pain bullying inflicts on victims, leading to emotional sharing and more positive actions [16, 41]. In other words, college students with high levels of virtuous personality are more likely to feel the emotions of the victim in bullying situations, moderate and alleviate negative emotions, and engage in positive behaviors to maintain interpersonal relationships, thereby performing more bystander defending behaviors. This suggests that schools and relevant authorities could enhance students’ virtuous personality traits through education and activities as part of prevention and intervention programs. Doing so would increase students’ ability to perceive others’ emotions and encourage bystanders to engage in more protective behaviors.

This study also found that moral identity mediates the effect of virtuous personality on bystander defending behavior among college students. Specifically, virtuous personality can both directly influence bystander defending behavior and exert an indirect effect through moral identity. On one hand, virtuous personality is closely linked to morality, reflecting unique moral content within the personality structure [16]. It is a product of the interaction between personality and morality, providing a bridge to further explore the relationship between personality traits and moral identity. The moral emotions triggered by virtuous personality can activate an individual’s internal moral identity, motivating them to engage in behaviors that align with their moral standards [14]. On the other hand, moral identity is a key intrinsic driver for moral behavior [19]. The gap between an individual’s current moral self-image and their ideal moral self-image creates psychological pressure, which activates the bystander’s moral identity regulation mechanism, prompting them to intervene positively in bullying situations [16]. When college students witness bullying, bystanders with high levels of virtuous personality are able to feel the pain of others and experience moral emotions such as sympathy and compassion [41]. Adolescents with strong moral identity are more likely to intervene positively in bullying situations [21], thereby engaging in bystander defending behavior. This also suggests that for college students with high levels of virtuous personality, moral cognitive factors—especially moral identity—should receive more attention in terms of their impact on adaptive development. Furthermore, this highlights the importance for schools and relevant authorities not only to enhance college students’ virtuous personality traits but also to provide opportunities for moral emotional communication and offer appropriate guidance in moral cognition. This can help encourage the occurrence of bystander defending behaviors.

Moreover, this study found that friendship quality moderates the first half of the mediation path through which virtuous personality affects bystander defending behavior via moral identity among college students. This is a statistically significant but small moderation effect, suggesting that high friendship quality has a minimal enhancing effect on the positive influence of virtuous personality on moral identity. For individuals with high friendship quality, virtuous personality enhances moral identity, which in turn increases bystander defending behavior. However, for individuals with low friendship quality, this mediating effect is weakened. In other words, friendship quality significantly strengthens the impact of virtuous personality on moral identity among college students. As the level of friendship quality increases, the effect of virtuous personality on moral identity also strengthens. This finding supports the theory of individual-environment interaction [21]. As two important factors influencing college students’ development, both virtuous personality and peer relationships not only affect development independently but also interact and influence each other, ultimately impacting student development. According to the “Protective Factor—Protective Factor Model,” the presence of one protective factor can enhance the positive effect of another [42]. Thus, as two important protective factors in college student development, the positive effects of virtuous personality and friendship quality not only accumulate but also interact, with high friendship quality amplifying the positive impact of virtuous personality on moral identity. This study, for the first time, examines the moderating mechanism of “virtuous personality → moral identity → bystander defending behavior” from the perspective of bystander behavior, integrating personality traits, cognition, and the environment into the study of bystander actions in bullying situations. This, to some extent, shifts the focus of bullying research. The analysis of the moderating effect reflects that the protective role of virtuous personality varies under different levels of friendship quality. On one hand, this indicates that the level of friendship quality has a significant impact on the development of college students’ mental health and changes in their behavioral attitudes. On the other hand, it suggests that, influenced by social development, cultural background, and personal experiences, the level of virtuous personality among college students needs further enhancement. Overall, virtuous personality plays a positive role in protecting against bullying, but this effect is also influenced and limited by other factors.

However, this study did not find evidence that friendship quality moderates the relationship between moral identity and bystander defending behavior. This lack of moderation in the second half of the pathway warrants further consideration. One possible explanation is that once an individual has developed a strong sense of moral identity, the direct influence of that identity on their behavior in a bullying situation may be relatively consistent, regardless of their friendship quality. In other words, a strong moral compass, once established, may guide behavior independently of immediate social context. Individuals with a high moral identity may feel a strong internal obligation to intervene, even if their friendships are not supportive or if they fear social repercussions [43]. Another potential explanation lies in the nature of the intervention behavior itself. Intervening in a bullying situation often requires a decisive and immediate response [44]. While friendship quality may influence the development of moral identity over time, the actual act of intervention may be driven more by deeply ingrained moral principles and a sense of personal responsibility that transcends immediate social considerations. Furthermore, the social pressure to conform or remain silent in bullying situations may be so strong that even individuals with supportive friendships may still hesitate to intervene, suggesting that moral identity is a more potent predictor of intervention than friendship quality at this stage. Future research should explore these possibilities further by examining the specific cognitive and emotional processes that mediate the relationship between moral identity and intervening behavior. It would also be valuable to investigate the role of other contextual factors, such as the severity of the bullying situation and the presence of authority figures, in moderating this relationship. Understanding why the second half of the pathway remains unregulated will provide a more complete picture of the complex interplay of factors that influence bystander intervention in bullying situations.

### Theoretical and practical significance

This study makes significant and original contributions to the field of moral psychology, particularly in understanding the interplay between virtuous personality, moral identity, and bystander intervention in bullying contexts. While prior research has explored related constructs, this study introduces a novel and integrative approach by operationalizing virtuous personality as a comprehensive antecedent variable and examining its specific mechanisms within a unique causal chain. Below are the study’s theoretical contributions and emphasize its originality in advancing existing knowledge. Unlike previous studies that have often treated virtuous personality as a broad independent variable influencing altruistic behavior [45], this study pioneers the use of virtuous personality as an integrative construct that encapsulates multidimensional virtues such as altruism, magnanimity, responsibility, integrity, benevolence, and kindness. By validating its role as a significant antecedent for both moral identity and bystander protective behavior, this study provides a more holistic understanding of how stable, integrative moral personality traits drive prosocial actions in bullying scenarios. This approach moves beyond merely establishing direct effects, offering a nuanced exploration of how virtuous personality fosters active intervention in contexts where inaction is prevalent, thereby refining our understanding of bystander engagement in complex moral landscapes. This study is the first to situate the moderating role of friendship quality within a causal chain originating from the integrative construct of virtuous personality. By doing so, it provides fresh empirical evidence and perspectives on how individual foundational traits interact with key environmental factors to shape moral behavior among college students. Unlike prior studies that has examined friendship quality in isolation or as a general social support factor, this study uniquely positions it as a moderator within a specific pathway, illuminating how it amplifies or constrains the translation of virtuous personality into bystander protective behavior through moral identity. This targeted investigation distinguishes our work from existing studies and underscores its originality in uncovering context-specific dynamics. Also, this study makes a groundbreaking contribution by engaging with ongoing debates about the independence of internalization and externalization dimensions within moral identity [46]. While some theories suggest that these dimensions operate independently through distinct pathways, this study proposes a more integrated process, positing that a strong internalized moral identity, reflected in virtuous personality traits, serves as a prerequisite for the externalization of moral values into active bystander intervention. This challenges the notion that externalization can occur without a robust internal foundation, offering a novel perspective that reframes the theoretical boundaries of moral identity. By demonstrating how virtuous personality facilitates intervention in challenging social environments, this study contributes to broader theoretical discussions about the mechanisms underlying moral action. In addition, this study provides a nuanced understanding of the conditions under which virtuous personality is most likely to translate into bystander intervention. Specifically, it highlights that the strength of an individual’s virtuous personality is particularly critical in situations where social norms discourage intervention or where the risks of intervening are high. This insight not only refines theoretical models of moral behavior but also informs practical interventions aimed at promoting bystander action and reducing bullying. By specifying these contextual boundaries, the study moves beyond generic associations between moral traits and behavior, offering a sophisticated framework for understanding how character development influences moral action in real-world settings.

From a practical standpoint, this study provides actionable insights for preventing and reducing bullying through educational and policy frameworks. Recognizing the protective role of virtuous personality, regardless of friendship quality, schools can integrate character education programs into their curriculum, focusing on virtues like empathy and responsibility through activities such as role-playing and community service [47]. At the policy level, schools can adopt and consistently enforce anti-bullying policies emphasizing bystander intervention and rewarding virtuous behavior, communicating these policies clearly and providing professional development for staff on fostering virtuous traits and positive peer relationships [48]. Encouraging participation in team-building activities and model-sharing can also enhance moral identity and bystander intervention, integrated into classroom lessons or extracurricular programs.

### Limitations and prospects

Although this study has shed light on the mechanisms through which virtuous personality influences bystander defending behavior among college students, there are still some limitations that require further improvement in future research. First, this study is cross-sectional in nature. While it is grounded in a solid theoretical framework, it does not allow for causal inferences and is more applicable to bystander defending behavior in online bullying. Future research could adopt intervention experiments or longitudinal tracking methods to test the moderated mediation model established in this study, while also exploring the causal relationship between virtuous personality and bystander defending behavior, as well as extending the longitudinal tracking design to real-life scenarios. Second, this study only focused on protectors within the bystander group, excluding helpers, outsiders, and reinforcers. Future research could consider examining the impact of virtuous personality on other types of bystanders, providing a deeper understanding of how virtuous personality influences bystander behavior and its underlying mechanisms. Additionally, while this study targeted college students, bullying incidents—especially school bullying—are most prevalent during adolescence [49]. Therefore, future research could focus on adolescent populations, expanding and enriching the sample, and further investigating the mechanisms behind bystander defending behavior. This would provide additional support for preventing and reducing bullying incidents.

## Conclusions

The main conclusions of this study are as follows: First, virtuous personality significantly and positively predicts bystander defending behavior; second, moral identity mediates the relationship between virtuous personality and bystander defending behavior; third, the indirect effect of virtuous personality on bystander defending behavior through moral identity is moderated by friendship quality.

## Data Availability

Data and materials are available on request from the corresponding author.
